# Practice day may be unnecessary prior to testing knee extensor strength in young healthy adults

**DOI:** 10.1080/23335432.2020.1766997

**Published:** 2020-05-28

**Authors:** Jamie E. Hibbert, Anthony S. Kulas, Patrick M. Rider, Zachary J. Domire

**Affiliations:** a Department of Kinesiology, East Carolina University, Greenville, NC, USA

**Keywords:** Dynamometry, strength testing, young adults, reliability

## Abstract

A practice session is common prior to strength testing. However, the benefits of practice have not been previously reported. The purpose of this study was to determine the effect of a practice session on peak torque, mean torque and between trial variability across three test days. We hypothesized that peak and mean torque would be higher and less variable the second and third test days than the first. Twenty-five healthy, young participants completed 3 maximal voluntary isometric and isokinetic knee extensions on three separate days. No difference in isometric torque was found between days 1 and 2, but there was a significant decrease in isokinetic torque (8.45 Nm). There was a significant decrease in both mean isometric and isokinetic torque from day 1 to day 3 (12.67 and 13.59 Nm). Contrary to our hypothesis, no benefit from a practice session was found. Healthy, young adults are able to produce peak knee extensor torques on the first day of testing and do not demonstrate any benefit from additional testing. Thus, a practice day preceding isometric and isokinetic knee extensor strength testing may not be necessary when testing healthy, young participants, and may, in fact, negatively impact subsequent strength measurements.

## Introduction

Maximal strength testing is commonly performed in both research and clinical settings as an assessment of muscle and joint function. Dynamometry has been used to assess both upper and lower extremity strength in healthy and pathological populations (May et al. 1997; Stackhouse et al. 2001; Martin et al. 2006; Miller et al. 2006). It has been adopted by clinicians as an objective strength measurement tool used to assess progress following injury or surgical intervention (Bohannon 1987). Dynamometry has been used successfully with many subsets of the population, including healthy young and older adults (Rantanen et al. 1997; Hartmann et al. 2009; Katoh and Isozaki 2014). Isokinetic dynamometry has also been used to determine input parameters for muscle modeling (Hatze 1981). Values for both isometric and isokinetic strength are often reported as they are easily tested and generalizable across populations and different movement patterns (Hatze 1981; Bohannon 1987; May et al. 1997; Rantanen et al. 1997; Stackhouse et al. 2001; Martin et al. 2006; Miller et al. 2006).

Strength testing is performed using some form of dynamometer typically either a hand-held, fixed or isokinetic dynamometer. There have been studies conducted that examined various types of dynamometers and found them to be both reliable and valid (Madsen 1996; Drouin et al. 2004; Shechtman et al. 2005; Hartmann et al. 2009; Katoh and Isozaki 2014). Isokinetic dynamometers are considered to be the gold standard for this type of measurement because of their accuracy and reliability (Stackhouse et al. 2001).

Despite the common use of isokinetic dynamometry to assess maximal strength there are other factors that may introduce variability in strength measurements when testing human participants such as sincerity of effort and familiarity with both the equipment and environment (Almosnino et al. 2011). As summarized in a review by Karni, acquisition of motor skills is a multifaceted process that typically improves with increased practice of the task being learned (Karni 1996). Therefore, researchers commonly bring participants in to the laboratory for a practice session prior to beginning true data collection for their study (Kues et al. 1994; Miller et al. 2006; Hartmann et al. 2009; Mayhew et al. 2009; Krupenevich et al. 2015). This is typically justified as a component of research methods as a means of allowing the participants to acclimate to the laboratory setting, the skill or talks being tested, the testing protocol, and the research team. This is especially important in studies utilizing a repeated measures design as a means to decrease test-retest bias.

As previously mentioned, the validity and reliability of dynamometry has been established, but these measurements do not take into account systematic improvements that could occur as a result of a learning effect. High day to day reliability can still be achieved even if there is a change in the average torque being produced if the two sessions are highly correlated. Standard Pearson correlations to determine the torque values from session to session demonstrate consistency in the measurement, but are not sensitive to systematic improvements (Weir 2005). Minimizing systematic changes due to a lack of familiarization is important because this type of strength testing is typically used during longitudinal intervention studies where the absolute change in torque is often used as a demonstration of the effect a given intervention on participant strength.

Few studies that examine validity and reliability of isokinetic dynamometry also report on systematic improvements, and no previous studies have examined systematic improvements as a primary objective (Symons et al. 2004; Sole et al. 2007; Hartmann et al. 2009; Park and Hopkins 2012; De Carvalho Froufe Andrade et al. 2013; Toonstra and Mattacola 2013). This information has been reported for hand-held measurement devices, but not isokinetic dynamometers which are considered the gold standard for this type of measurement (Katoh and Isozaki 2014; Hansen et al. 2015; Ruschel et al. 2015). Two studies have reported means for strength testing on different days, but have not statistically tested for differences (Park and Hopkins 2012; Toonstra and Mattacola 2013). However, both of these studies present small increases with repeat strength testing. Four studies have examined systematic bias across multiple days of strength testing with mixed results. De Carvalho Froufe Andrade and colleagues report no systematic bias across two testing days in isometric, concentric, and eccentric testing of the knee flexors and extensors (de Carvalho Froufe Andrade et al. 2013). Symons and colleagues examined knee extensors isometric, concentric, and eccentric strength across two testing sessions and reported a small (6.7%), but significant increase in eccentric torque on day two (Symons et al. 2004). Hartmann and colleagues conducted a practice session, for which they did not present data, and two additional testing sessions testing knee concentric flexion and extension at 60°/second and 120°/second, as well as ankle plantarflexion and dorsiflexion at 60°/second. There was a small, but significant increase in knee flexion concentric strength at 120°/second, but no difference at the slower speed or in other movements tested (Hartmann et al. 2009). Sole and colleagues tested concentric and eccentric knee flexion and extension on two days. They found a significant increase in knee extensor torque on the second day (Sole et al. 2007). The mixed results from these studies provide some evidence for the implementation of a practice session prior to strength testing, but further research is needed to provide clarification. Furthermore, it is unclear if an additional practice session would be valuable since the aforementioned studies utilized only two testing days.

The purpose of this study was to determine the effect of a practice session on both isometric and isokinetic knee extensor peak torque, mean torque and between trial variability across three test days. We hypothesized that both isometric and isokinetic knee extensor peak torque and mean torque would be higher and less variable the second and third test days than on the first (practice) day.

## Methods

Twenty-five healthy, young (age 21.3 ± 3) participants (13 males, 12 females) were recruited to complete the study. All participants were free from musculoskeletal injury and reported no pain with activities of daily living. This number of participants is enough to provide more than 80% power with an effect size of approximately 0.6. The University Institutional Review Board approved all experimental procedures, and written informed consent was obtained from each participant prior to the start of testing. Each participant reported to the biomechanics lab for maximal isometric and isokinetic strength testing three times. The three visits were completed within 7 days of beginning the protocol. Participants were encouraged to schedule their test sessions on non-consecutive days when possible. There was a minimum of 24 hours between each testing session. Only one participant completed all three testing sessions on consecutive days. The average number of days between testing sessions 1 and 2 was 1.8 ± 0.7 days. The average number of days between testing sessions 2 and 3 was 2.0 ± 1.2 days.

**Figure 1.**
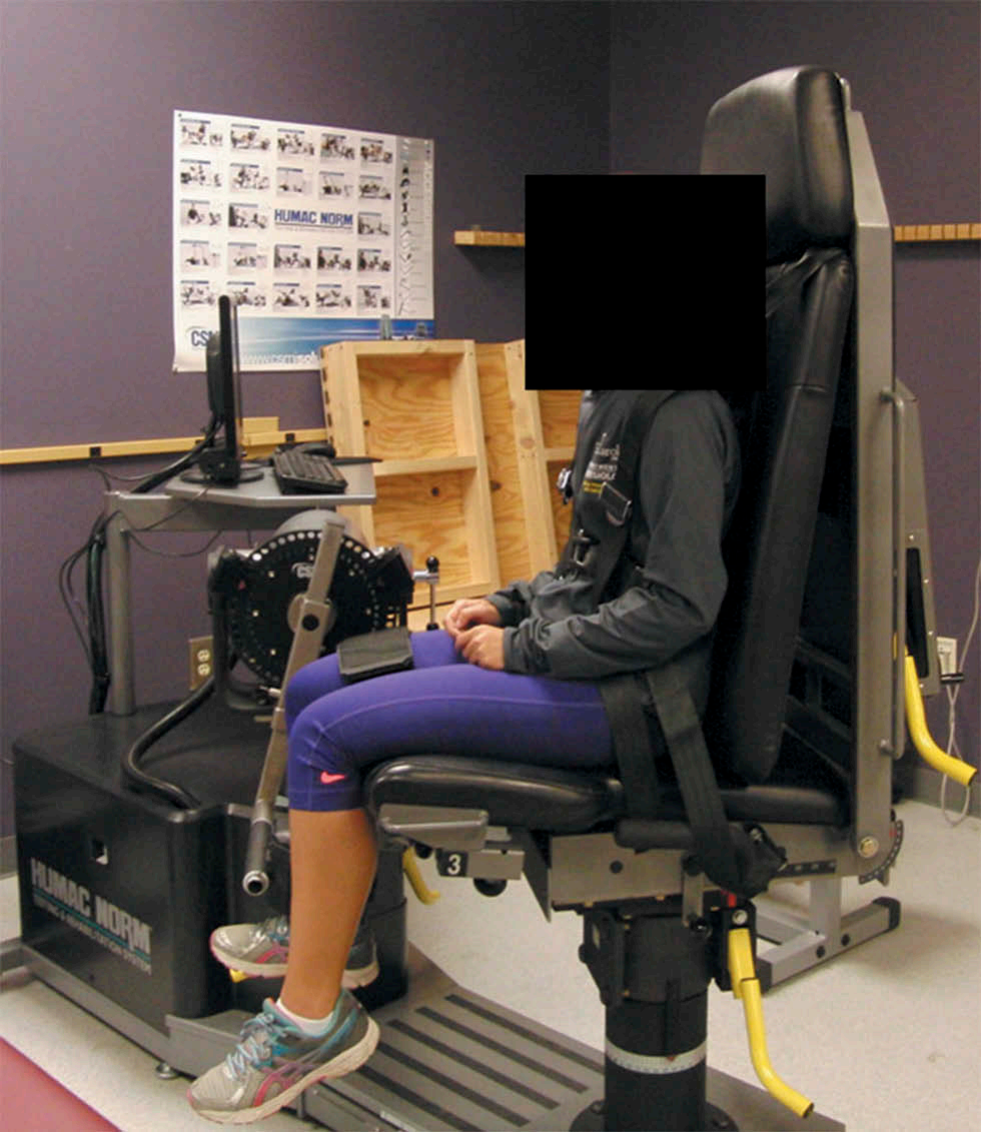
Experimental setup for isometric knee extensor strength testing

Prior to each testing session, participants completed a 5-minute warm-up on a cycle ergometer. Participants were given two submaximal trial repetitions prior to the test each day to ensure proper dynamometer setup. All participants were seated with an upright torso. The participant was positioned such that both hip and knee angles were at 90° of flexion (Figure 1). Participants were instructed to perform these repetitions at 50% of their maximal effort. Participants then completed 3 maximal voluntary isometric contractions (MVICs) of the right knee extensors using a HUMAC isokinetic dynamometer (HUMAC NORM Testing & Rehabilitation System, CSMI Medical Solutions, Stoughton, MA). Each contraction was held for 5 seconds. Participants were allowed no less than 5 seconds between repetitions. Following the completion of the isometric contractions, the participants performed 3 maximal voluntary isokinetic contractions at 60 °/second through 90 degrees of knee extension. Participants began each repetition with the knee flexed to 90 degrees and completed the repetition when the knee was fully extended. As with isometric testing, prior to completing the maximal contractions, the participants were allowed 2 warm-up repetitions (50% of their maximal effort) to ensure proper dynamometer setup. Participants were instructed to extend their knee as quickly as possible. No less than 5 seconds of rest were allowed between repetitions. Consistent verbal encouragement by the study team was provided during each testing session. Participants were also allowed to view the monitor to get visual feedback on their torque production.

Both isometric and isokinetic data collected were analyzed for peak torque produced by each subject on each day as well as the mean torque produced over the three maximal trials. Mean torque was calculated as the mean of the peaks produced in each trial. The standard deviation of the peak torques from each repetition produced for each day was also calculated to determine the variability between trials for each participant.

The values for the peak torque, mean torque, and standard deviation for both isometric and isokinetic testing were compared across the three testing days using repeated measures ANOVAs with Bonferonni corrections for multiple comparisons. In consideration that the present study’s sample was approximately evenly mixed between males and females, we performed an initial 3 × 2 RMANOVAs with sex as a between factor to determine if males and females respond to the testing days differently implying a sex*day interaction. Overall, while main effects for sex were present across all variables with males being ~30% stronger than females, there were no sex*day interactions. Further, the sex differences were absent after re-running the analyses with the torques normalized to mass. Given the absence of sex*day interactions and that subject mass accounted for the sex differences, we pooled the data to run the repeated measures ANOVAs. A p-value of 0.05 was set as the cut off for determining statistical significance. P-values between 0.05 and 0.10 were identified as trends and indicates there may be a true effective difference (Curran-Everett and Benos 2004). In the case of trends, we reported Cohen’s *d_z_* effect sizes to determine the magnitude of differences between days (Lakens 2013). Cohen’s *d_z_* is appropriate for within subject designs, takes into account the correlation between two measures, and is calculated as $\frac{t}{\sqrt{n}}$ where t equals the t-value from a follow-up paired samples t-test and n represents the number of participants (Lakens 2013). By convention, effect sizes were interpreted as: 0.20–0.49 (small/weak), 0.50–0.79 (medium/moderate), 0.80–1.29 (large/strong), and > 1.30 (very large/very strong) (Rosenthal 1996). SPSS statistical analysis software was used to calculate the trial mean squared (TMS), error mean square (EMS), and between mean square (BMS) which come from the repeated measures ANOVAs. The TMS, EMS, and BMS, are the three sources of error used to calculate the Intraclass Correlation Coefficients (ICCs). The ICC (2,k) formula was used to assess the similarity of mean isometric and isokinetic torques across the testing days while the ICC (2,1) formula was used to assess the similarity of peak isometric and isokinetic torques across the three testing days (Koo and Li 2016). By convention, ICCs between 0.5 and 0.75, between 0.75 and 0.90, and greater than 0.90 were considered moderate, good, and excellent reliability respectively (Koo and Li 2016). The precision of the measurements across days was reported as the standard error of the measurement (SEM) and calculated as: $SEM=SD\times \surd \left(1-ICC\right)$ (Beaton 2000). While the repeated measures ANOVAs tested for significant differences between days of testing, significance testing using ANOVAs do not provide measures of similarity and precision like the ICC and SEM do respectively. In addition, to complement the ICCs and SEMs, we also calculated the minimal detectable difference (MDD_95_) which creates a 95% confidence interval using the SEM to identify the lowest change/difference that could confidently be considered as exceeding measurement error and therefore a ‘real’ change. The formula for MDD_95_ used was $1.96\times \sqrt{2}\times SEM$ (Beaton 2000).

## Results

### Isometric torques

Table 1 reports the means, standard deviations, and 95% confidence intervals for the peak, mean, and standard deviation for isometric torques. There were no significant differences in peak isometric torques (p = 0.255), mean torques (p = 0.079), and standard deviations (0.116) across the 3 testing days. A trend (0.10 > p > 0.05) was identified from the mean torque analysis. Cohen’s d_z_ effect sizes were: 0.16 (day 1 – day 2), 0.50 (day 1 – day 3), and 0.28 (day 2 – day 3).

**Table 1.** Average values for peak isometric torque, mean isometric torque and standard deviation showing variability of peak isometric torque between trials on each of the three test days. All values are shown as the mean of that variable ± SD. * p <.05 comparing day 1 to day 3. All other day-to-day comparisons were non-significant

	Peak Torque (N m)	Mean Torque (N m)	Standard Deviation (N m)
Day 1	216.32 ± 55.76	207.59 ± 53.82*	8.79 ± 4.41
Day 2	212.40 ± 66.81	203.04 ± 63.16	9.63 ± 6.06
Day 3	206.56 ± 60.28	194.92 ± 57.76	11.47 ± 5.01

**Figure 2.**
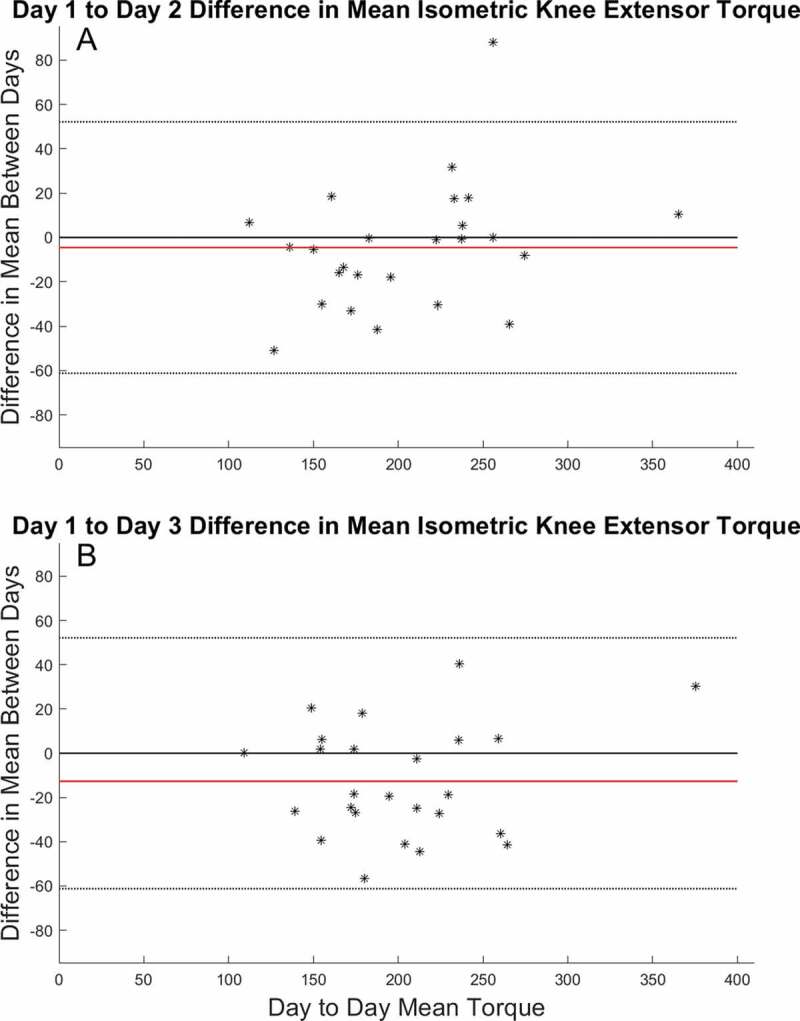
(a) Comparison of the difference in isometric mean values from day 1 to day 2 (b) Comparison of the difference in isometric mean values from day 1 to day 3. Each data point represents the difference in mean isometric torque production values for each individual participant. The red line represents the overall mean difference between the two days. The dotted lines represent the upper and lower limits of agreement

The mean isometric torque ICC across testing days was 0.96 with a SEM of 11.20 Nm. The minimal detectable difference (MDD_95_) across days was 31.03 Nm. The SEM and MDD_95_ represented 5.39% and 14.90% of the mean measurement respectively. The peak isometric torque ICC across testing days was 0.88 with an SEM of 19.03 Nm. The MDD_95_ across days was 52.76 Nm. The SEM and MDD_95_ represented 8.80% and 24.40% of the mean measurement respectively. This can also be seen from the Bland-Altman plots of the data (Figure 2).

### Isokinetic torques

Table 2 reports the means, standard deviations, and 95% confidence intervals for the peak, mean, and standard deviations for isokinetic torques. Peak isokinetic torques were significantly different across testing days (p = 0.007). Pairwise comparisons revealed that peak torque was significantly on day 1 compared to days 2 (p = 0.031) and 3 (p = 0.028). There were no other day-to-day differences. Mean isokinetic torques were significantly different across testing days (p = 0.003). Pairwise comparisons showed day 1 produced higher torques compared to day 3 (p = 0.012). There were no other day-to-day differences. There was a trend (p = 0.82) for the standard deviations across testing days. Cohen’s d_z_ effect sizes were: 0.17 (day 1 – day 2), 0.42 (day 1 – day 3), and 0.28 (day 2 – day 3).

**Table 2.** Average values for peak isokinetic torque, mean isokinetic torque and standard deviation showing the variability of peak isokinetic torque between trials on each of the three test days. All values are shown as the mean of that variable ± SD. * p <.05 comparing day 1 to day 2 and day 1 to day 3. All other day-to-day comparisons were non-significant

	Peak Torque (N m)	Mean Torque (N m)	Standard Deviation (N m)
Day 1	187.87 ± 51.36*	178.19 ± 50.93*	9.70 ± 12.05
Day 2	178.17 ± 47.53	170.54 ± 46.26	7.64 ± 4.82
Day 3	173.68 ± 44.59	164.60 ± 44.17	9.77 ± 6.21

**Figure 3.**
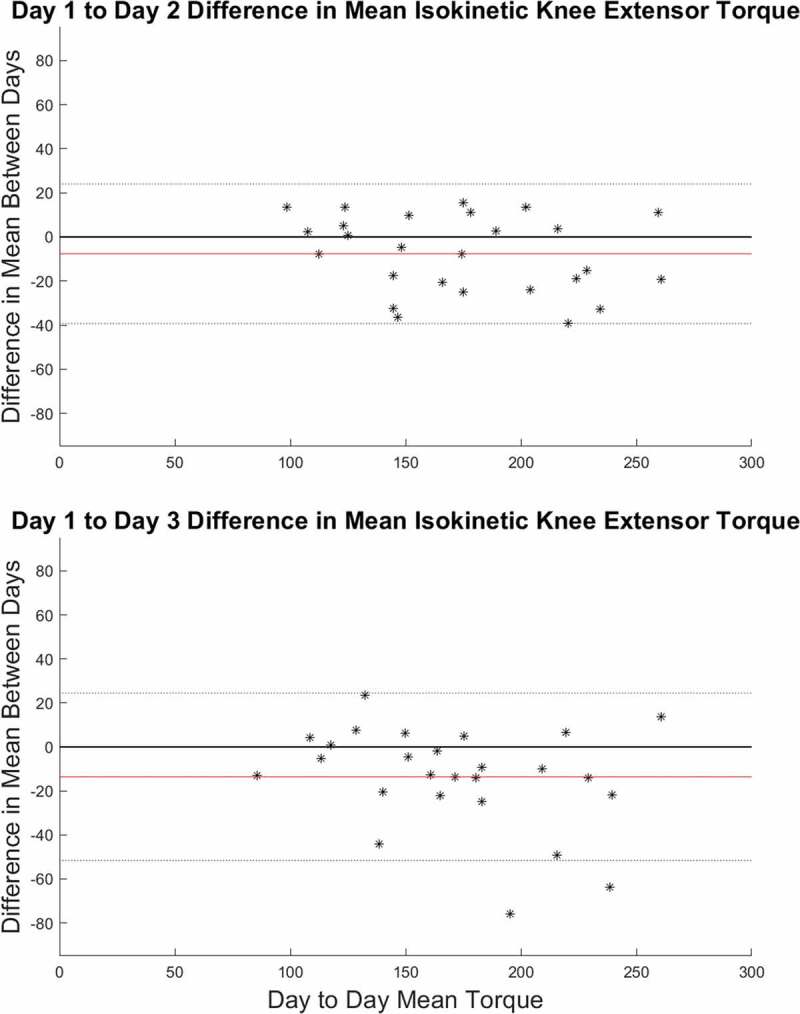
(a) Comparison of the difference in isokinetic mean values from day 1 to day 2 (b) Comparison of the difference in mean isokinetic values from day 1 to day 3. Each data point represents the difference in mean isokinetic torque production values for each individual participant. The red line represents the overall mean difference between the two days. The dotted lines represent the upper and lower limits of agreement

The mean isokinetic torque ICC across the three testing days was 0.95 with a SEM of 10.81 Nm. The MDD_95_ across days was 29.95 Nm. The SEM and MDD_95_ represent 6.00% and 16.6% of the mean isokinetic torques respectively. The peak isokinetic torque ICC across the three testing days was 0.88 with a SEM of 17.91 Nm. The MDD_95_ across days was 49.66 Nm. The SEM and MDD_95_ represent 9.54% and 26.40% of the peak isokinetic torques respectively. This can also be seen from the Bland-Altman plots of the data (Figure 3).

## Discussion

The current results call into question whether an individual familiarization session is needed in order to ascertain maximal voluntary strength. We hypothesized that if a familiarization session is needed, peak strength would increase on days 2 and 3 compared to the day 1 familiarization session. Rather than an increase in torque production from day 1 to days 2 and 3, day 1 actually exhibited the highest peak isokinetic torques compared to days 2 and 3. In addition, the mean isokinetic torque was significantly higher on day 1 compared to day 3 with no significant difference between days 1 and 2. In regard to the isometric torques across testing days, while there were no significant differences for peak isometric torques, there was a trend for mean isometric torque with day 1 showing a moderate effect size towards a greater torque compared to day 3 and low effect size compared to day 2. Overall, the results for both isokinetic and isometric torques suggest that maximal voluntary strength can be attained in a single session without having to have a dedicated familiarization session first.

Isokinetic dynamometers are considered the gold standard for strength measurement because they have been extensively validated (Drouin et al. 2004). Many of the previously published studies examining validity and reliability of dynamometry to measure both isometric and isokinetic torque were conducted to validate new devices (May et al. 1997; Shechtman et al. 2005; Laurent et al. 2010; Martin et al. 2006; Toonstra and Mattacola 2013; Hansen et al. 2015; Ruschel et al. 2015). These studies are very important, but rather than test for systematic changes they correlate the torque measured with the new device to that measured by the established standard. Other studies reported only correlation data for the trial sessions (Rantanen et al. 2003; Shechtman et al. 2005; Martin et al. 2006). Correlation data is useful for determining consistency, but not determining the presence or absence of any learning effect where a familiarization session would benefit. The high ICCs and low SEMs found in the current study are generally in agreement with past work validating strength measurements.

As described in the introduction, several studies have previously reported the systematic bias of strength measurements between two test days. It has been reported that there was a significant difference in knee flexion strength at 120°/second, (Hartmann et al. 2009) knee extension at 90°/second, (Symons et al. 2004) concentric knee extension at 60°/second,(Sole et al. 2007) or no differences between testing sessions (de Carvalho Froufe Andrade et al. 2013). Given that the differences reported occurred across studies that used different testing parameters there is a possibility that the differences are present or absent base on the protocol utilized. However, it is worth noting that two of these studies measured differences in older adults which are different than the population tested in the present study (Symons et al. 2004; Hartmann et al. 2009).

Additional studies have sought to examine differences between testing sessions, but rather than examine day-to-day reliability they conducted all of the testing sessions on the same day with only a short break between sessions (May et al. 1997; Shechtman et al. 2005; de Carvalho Froufe Andrade et al. 2013; Katoh and Isozaki 2014; Hansen et al. 2015). This is different than the current study because we were interested in the necessity of a practice day, not practice repetitions on the same day as the testing session. Day-to-day reliability and precision of muscle strength is critically important for determining meaningful changes in strength in longitudinal intervention studies.

The lack of improvement observed with the task performed in this study may be related to knee extension being a very familiar task to a young healthy population. This idea is supported by a study by Roemmich and Bastian that indicated that faster relearning of a motor skill is influenced by previous exposure (Roemmich and Bastian 2015). It may be that the two warm-up repetitions allowed at the beginning of each testing session were enough of a re-exposure to the movement pattern that the participant was able to perform maximally on the first day of testing that was originally designed to be a session completely for practice.

It is possible that lingering fatigue from the previous sessions might explain the overall trends of muscle strength decreasing despite a relatively low load compared to other studies focusing on fatigue (Rodacki et al. 2002; Pincivero et al. 2003; Pethick et al. 2015). The participants performed three, five second maximal voluntary isometric contractions and three maximal voluntary isokinetic contractions during each visit. The trend towards an increase in variability observed across the testing sessions may also indicate that maximal effort was not achieved. However, the effect size showing this change from day 1 to day 3 was only 0.42 (Cohen’s d_z_) and is considered a small, and potentially unimportant, effective difference. Nevertheless, we cannot discount the possibility that participants were fatigued on subsequent testing sessions where day three knee extensor torques were collectively ~4.5–7.5% lower than day one. While future research should aim to identify the sources of this fatigue i.e. physical or mental, the results of the current study support the use of day one strength measurements to approximate a participant’s maximal voluntary knee extensor strength.

Some limitations of the current study are that the testing is only in the knee extensor muscle group in an isolated manner. Results may be different if the task were completed in a different muscle group or in a different mode of testing (Ploutz-Snyder and Giamis 2001). However, isolated isometric and isokinetic testing are common strength testing techniques that are widely used (Madsen 1996; Rantanen et al. 2003; Shechtman et al. 2005; Martin et al. 2006; Park and Hopkins 2012; de Carvalho Froufe Andrade et al. 2013). Another limitation of this study is that it was conducted in young healthy adults. These results may not be generalizable to other populations, such as children or older adults (Ploutz-Snyder and Giamis 2001; Fagher et al. 2016).

The results of this study have the potential to change the way that researchers design studies, which has the potential to save time, effort and money. These results could also have clinical implications for clinicians who employ isometric dynamometry for strength testing measures. If these individuals observe improvements in torque production of their patients, they can be more confident that the improvements are true rather than a result of familiarity with the equipment and testing procedure.

Based on the data collected, healthy, young adults are able to produce peak isometric knee extensor torques on the first day of strength testing that are not different or larger from torques produced on the second day and third day. These results indicate that when young, healthy participants are involved in a study it is not necessary to have a practice day prior to beginning maximal isometric strength testing. Particularly if done for multiple days, practice procedures could actually negatively impact maximal torque production. The two sub-maximal practice trials performed in this study seem to be sufficient to eliminate any additional learning effect.
